# Supplementary material for: Content-rich biological network constructed by mining PubMed abstracts
Source: BMC Bioinformatics. 2004 Oct 8;5:147. doi: 10.1186/1471-2105-5-147 (PMC528731; doi:10.1186/1471-2105-5-147)
Supplement: Additional File 2 — The original results of the above study (non-essential files are deleted to keep the file size under the limit set by BMC bioinformatics). [file 1471-2105-5-147-S2.bz2 › chilibotAdditionalFile2/dip20/450ID10477718E10461/html/CCL5_CCR5.html]

 


 **CCL5** and **CCR5** 
  
Found 460 abstracts in PubMed, retrieved 20.  
 

 What does Google say? 
 PDF only 
| .edu only 

---

**Interactive relationship** (e.g. stimulation, inhibition, etc)

**Inhibitory relationship**- Glycosaminoglycans removal from the cells by glycosaminidases treatment prevented RANTES  [ **CCL5** ]  binding to syndecan 1 and syndecan 4 and decreased RANTES  [ **CCL5** ]  binding to  **CCR5**  on the  **CCR5**  positive HeLa cells.  Ref: 12773479 Glycobiology< MedlineTA>Glycobiology,
- CONCLUSIONS Our results demonstrate that an interaction of HGV E2 with CD81 leads to increased RANTES  [ **CCL5** ]  secretion and decreased  **CCR5**  surface expression.  Ref: 12824783 AIDS, 2003
- The chemotactic response of  **CCR5**  CAM T82P to RANTES  [ **CCL5** ]  was similar to that of  **CCR5**  WT, but  **CCR5**  CAM T82K was dramatically decreased.  Ref: 12837756 J Biol Chem< MedlineTA>J Biol Chem, 2003
- Ad35K infected cell culture medium, containing recombinant 35K, potently reduced migration of  **CCR5**  transfected 293 cells by 95% in response to the CC chemokine RANTES  [ **CCL5** ] , but had no effect on cells transfected with the CX3CR1 fractalkine receptor.  Ref: 12777060 Immunobiology, 2003
**Neutral relationship**- This complex is likely a functional unit in which PGs may modulate RANTES  [ **CCL5** ]  binding to  **CCR5** .  Ref: 12773479 Glycobiology< MedlineTA>Glycobiology, 2003
- P2 resembles AOP RANTES  [ **CCL5** ]  in that it is a superagonist of  **CCR5**  and potently induces receptor sequestration.  Ref: 12767983 J Virol, 2003
- Confocal microscopy analysis as well as co immunoprecipitation experiments show a RANTES  [ **CCL5** ]  independent heteromeric complex, which comprises on the  **CCR5**  positive HeLa cells, syndecan 1, syndecan 4 and  **CCR5** .  Ref: 12773479 Glycobiology< MedlineTA>Glycobiology, 2003
- **CCR5**  is a G protein coupled receptor for RANTES  [ **CCL5** ] , MIP 1a, MIP 1b and MCP 2 that functions as the front line coreceptor for human immunodeficiency virus type 1 infection.  Ref: 12837756 J Biol Chem< MedlineTA>J Biol Chem, 2003
- However, the remaining expression of CCR1 and  **CCR5**  correlated reciprocally with an ongoing unresponsiveness to the CCR1 and  **CCR5**  agonists CCL3 macrophage inflammatory protein 1alpha MIP 1alpha, CCL4 MIP 1beta, and  **CCL5**  regulated on activation, normal T expressed and secreted RANTES  [ **CCL5** ] , all chemokines binding to these two receptors.  Ref: 12885942 J Leukoc Biol, 2003
- Co immunoprecipitation studies demonstrate that RANTES  [ **CCL5** ]  forms complexes with glycanated syndecan 1 and syndecan 4, in addition to  **CCR5**  on the  **CCR5**  positive HeLa cells.  Ref: 12773479 Glycobiology< MedlineTA>Glycobiology, 2003
- However, the specific functional relevance of RANTES  [ **CCL5** ]  oligomerization for distinct steps of leukocyte recruitment on inflamed endothelium mediated by the RANTES  [ **CCL5** ]  receptors CCR1 and  **CCR5**  remains undefined.  Ref: 12763925 Blood< MedlineTA>Blood, 2003
- This interaction induces a dose dependent release of RANTES  [ **CCL5** ]  and down regulation of  **CCR5**  surface expression with concomitant intra cellular accumulation of  **CCR5**  proteins.  Ref: 12824783 AIDS, 2003
- The present findings suggest that MCP 1, RANTES  [ **CCL5** ] , and IP 10 may participate in the pathogenesis of mercuric chloride induced tubulointerstitial fibrosis with mononuclear cell infiltration, via CCR2, CCR1 or  **CCR5** , and CXCR3, respectively.  Ref: 12834626 Exp Mol Pathol, 2003
- Whereas the amplitude of calcium mobilization induced by RANTES  [ **CCL5** ]  stimulation was lower in the  **CCR5**  CAM than the wild type WT receptor, MCP 1 induced a higher signal in the CCR2 CAM than in CCR2 WT.  Ref: 12837756 J Biol Chem< MedlineTA>J Biol Chem, 2003
- Therefore, RANTES  [ **CCL5** ]  bindings to syndecan 1 and syndecan 4 depend from glycosaminoglycans and facilitate RANTES  [ **CCL5** ]  interaction with  **CCR5** .  Ref: 12773479 Glycobiology< MedlineTA>Glycobiology, 2003
- Enhanced protein concentrations of RANTES  [ **CCL5** ] , MIP 1a, and MIP 1beta were present in WG lung lesions, indicating redundancy of ligands for  **CCR5**  in affected tissue.  Ref: 12858455 J Rheumatol, 2003

**Non-interactive relationship** (e.g. studied together, co-existance, homology, etc.)

- KA treatment did not affect mRNA levels for the macrophage inflammatory protein 1alpha MIP 1alpha or the regulated upon activation normal T cells expressed and secreted protein RANTES  [ **CCL5** ]  in either wild type or  **CCR5**  KO mice.  Ref: 12807425 J Neurochem, 2003
- Potential molecular differences between early and late rejection grade > =2 episodes were examined by testing chemokine and receptor gene expression.METHODS Expression of inducible protein IP 10, monokine induced by IFN gamma Mig, interferon inducible T cell alpha chemoattractant I TAC, regulated on activation normal T cell expressed and secreted RANTES  [ **CCL5** ] , and their receptors CXCR3 and  **CCR5**  was tested in 60 endomyocardial biopsies from 24 patients using quantitative Taqman real time polymerase chain reaction PCR.  Ref: 12829909 Transplantation, 2003
- Notably, the formation of higher order RANTES  [ **CCL5** ]  oligomers was crucial for CCR1 mediated arrest but not for  **CCR5**  mediated spreading transmigration in flow or transendothelial chemotaxis of leukocytes.  Ref: 12763925 Blood< MedlineTA>Blood, 2003
- Furthermore, aminoxypentane RANTES  [ **CCL5** ]  abolishes virus replication, suggesting that these SIV strains utilize the chemokine receptor  **CCR5**  for entry into astrocytes.  Ref: 12768005 J Virol, 2003
- Furthermore, the presence of  **CCL5**  and CXCL10 in the cytolytic granules of tissue infiltrating CD8 T cells expressing  **CCR5**  and CXCR3 reveals a potential self recruiting mechanism involving activated effector cytotoxic T cells.  Ref: 12819030 Am J Pathol, 2003
- We show that the CC chemokine, RANTES  [ **CCL5** ] , binds not only to high affinity binding sites on  **CCR5**  positive HeLa cells, but also to low affinity binding sites on HeLa cells, expressing or lacking RANTES  [ **CCL5** ]  G protein coupled receptors.  Ref: 12773479 Glycobiology< MedlineTA>Glycobiology, 2003
- P1, while less potent than P2, has the advantage of significantly reduced signaling activity via  **CCR5**  30% of that of RANTES  [ **CCL5** ] .  Ref: 12767983 J Virol, 2003
- After 2 to 4 weeks, L. amazonensis infected mice had significantly delayed and depressed expression of inflammatory cytokines interleukin 12 IL 12, gamma interferon, IL 1alpha, IL 1beta, CC chemokines CC chemokine ligand 3 CCL3 macrophage inflammatory protein 1alpha MIP 1alpha, CCL4 MIP 1beta,  **CCL5**  RANTES  [ **CCL5** ] , MIP 2, and chemokine receptors CCR1, CCR2,  **CCR5**  in foot tissues and draining lymph nodes compared to the expression in L. major infected controls.  Ref: 12874303 Infect Immun, 2003
- Oligomerization of RANTES  [ **CCL5** ]  is required for CCR1 mediated arrest but not  **CCR5**  mediated transmigration of leukocytes on inflamed endothelium.  Ref: 12763925 Blood< MedlineTA>Blood, 2003
- In conclusion, RANTES  [ **CCL5** ]  and  **CCR5**  represent potential mediators of 1 HSC migration and proliferation and 2 a cross talk between HSCs and leukocytes during fibrogenesis.  Ref: 12829440 Am J Physiol Gastrointest Liver Physiol< MedlineTA>Am J Physiol Gastrointest Liver Physiol, 2003
- Infiltrating CD8 T Cells in Oral Lichen Planus Predominantly Express  **CCR5**  and CXCR3 and Carry Respective Chemokine Ligands RANTES  [ **CCL5** ]   **CCL5**  and IP 10 CXCL10 in Their Cytolytic Granules A Potential Self Recruiting Mechanism.  Ref: 12819030 Am J Pathol, 2003
- Human Hepatic Stellate Cells express  **CCR5**  and RANTES  [ **CCL5** ]  to induce proliferation and migration.  Ref: 12829440 Am J Physiol Gastrointest Liver Physiol< MedlineTA>Am J Physiol Gastrointest Liver Physiol, 2003
- Furthermore, infiltrating T cells, particularly CD8 T cells, were positive for RANTES  [ **CCL5** ]   **CCL5**  and IP 10 CXCL10, the ligands of  **CCR5**  and CXCR3, respectively.  Ref: 12819030 Am J Pathol, 2003
- Removal of glycosaminoglycans by glycosaminidases treatment of the complexes, RANTES  [ **CCL5** ]  syndecan 1 syndecan 4  **CCR5** , immobilized on beads, reversed syndecan 1 and syndecan 4 bindings.  Ref: 12773479 Glycobiology< MedlineTA>Glycobiology, 2003
- **CCR5**  D32 and RANTES  [ **CCL5** ]  28 and 401 polymorphisms were determined by either specific primers or direct sequencing.  Ref: 12858455 J Rheumatol, 2003
- The chemokine CC chemokine ligand CCL 5  [ **CCL5** ]  RANTES  [ **CCL5** ]  as well as its respective receptor  **CCR5**  mediate leukocyte infiltration during inflammation and are up regulated early during the course of glomerulonephritis GN.  Ref: 12759447 J Immunol, 2003
- As well as potently inhibiting the entry of  **CCR5**  using HIV 1 strains, RANTES  [ **CCL5** ]  can enhance HIV 1 infectivity under certain experimental conditions.  Ref: 12714503 Blood< MedlineTA>Blood, 2003
- The biopsies were taken in the first 3 months or from the 9th to the 12th month following transplantation.RESULTS IP 10, Mig, RANTES  [ **CCL5** ] , CXCR3, and  **CCR5**  expression levels were increased in the later versus earlier biopsies P< ; =0.01 despite no change in histologic rejection grade status.CONCLUSION These results demonstrate significantly increased expression of T cell chemoattractants in heart allografts during later rejection when compared with episodes occurring shortly after transplantation.  Ref: 12829909 Transplantation, 2003
- METHODS Lung biopsies from 4 patients that had classical features of WG were examined for protein expression of  **CCR5** , RANTES  [ **CCL5** ] , MIP 1a and MIP 1beta using immunohistochemistry.  Ref: 12858455 J Rheumatol, 2003
